# Clinical significance and prognostic role of hypoxia-induced microRNA 382 in gastric adenocarcinoma

**DOI:** 10.1371/journal.pone.0223608

**Published:** 2019-10-09

**Authors:** An Na Seo, Yukdong Jung, Hyeonha Jang, Eunhye Lee, Han-Ik Bae, Taekwon Son, Ohkyung Kwon, Ho Young Chung, Wansik Yu, You Mie Lee

**Affiliations:** 1 Department of Pathology, School of Medicine, Kyungpook National University, Daegu, South Korea; 2 Department of Pathology, Kyungpook National University Chilgok Hospital, Daegu, South Korea; 3 BK21 Plus KNU Multi-Omics Based Creative Drug Research Team, College of Pharmacy, Kyungpook National University, Daegu, South Korea; 4 Research Institute of Pharmaceutical Sciences, College of Pharmacy, Kyungpook National University, Daegu, South Korea; 5 Research Institute of Pharmaceutical Sciences, College of Pharmacy, Seoul National University, Seoul, South Korea; 6 Department of Surgery, School of Medicine, Kyungpook National University, Kyungpook National University Chilgok Hospital, Daegu, South Korea; 7 Department of Surgery, School of Medicine, Kyungpook National University, Kyungpook National University Hospital, Daegu, South Korea; Inha University Hospital, REPUBLIC OF KOREA

## Abstract

Hypoxia and angiogenesis are critical components in the progression of solid cancer, including gastric cancers (GCs). miR-382 has been identified as a hypoxia-induced miR (hypoxamiR), but the clinical significance in GCs has not been identified yet. To explore the clinical and prognostic importance of miR-382 in GCs, the surgical specimens of 398 patients with GCs in KNU hospital in Korea, the total of 183 patients was randomly selected using simple sampling methods and big data with 446 GCs and 45 normal tissues from the data portal (https://portal.gdc.cancer.gov/) were analysed. Expression of miR-382 as well as miR-210, as a positive control hypoxamiR by qRT-PCR in histologically malignant region of GCs showed significantly positive correlation (*R* = 0.516, *p*<0.001). High miR-210 and miR-382 expression was significantly correlated with unfavorable prognosis including advanced GCs (AGC), higher T category, N category, pathologic TNM stage, lymphovascular invasion, venous invasion, and perinueral invasion, respectively (all *p*<0.05). In univariate analysis, high miR-210 expression was significantly associated with worse overall survival (OS) (*p* = 0.036) but not high miR-382. In paired 60 gastric normal and cancer tissues, miR-382 expression in cancer tissues was significantly higher than normal counterpart (*p* = 0.003), but not miR-210 expression. However, by increasing the patient number from the big data analysis, miR-210 as well as miR-382 expression in tumor tissues was significantly higher than the normal tissues. Our results suggest that miR-382, as novel hypoxamiR, can be a prognostic marker for advanced GCs and might be correlated with metastatic potential. miR-382 might play important roles in the aggressiveness, progression and prognosis of GCs. In addition, miR-382 give a predictive marker for progression of GCs compared to the normal or preneoplastic lesion.

## Introduction

Gastric cancer (GC) is the fifth most common type of cancer and the third leading cause of cancer related deaths worldwide [1]. Worldwide mortality rates for patients with GC have declined significantly in recent decades, mainly due to earlier diagnosis, improved surgical techniques, and better adjuvant treatment. However, the survival rate of patients with advanced GC (AGC) remains very poor because the majority of such patients still present with metastatic or recurrent disease, thereby requiring systemic treatment. While there has been a modest improvement in cytotoxic chemotherapy regimens for AGC and a slight increase in overall survival (OS) [2], the sensitivity to treatment differs in individual patients. In addition, molecular targeted agents have been actively investigated and tested in AGC during the past decade. To date, only two classes of agents have been demonstrated to be effective: a humanized anti-HER2 antibody and anti-vascular endothelial growth factor receptor (VEGFR) antibody, and tyrosine kinase inhibitors [3]. The reason that GC responds poorly to chemotherapeutic and specific targeting agents remains unknown. Accumulating data suggest that tumor hypoxia might contribute to failures of radiotherapy and chemotherapy, resulting in unfavorable prognoses in patients with various malignancies [4–7]. Adaptation of tumor cells to hypoxic conditions has significant biological effects and contributes to tumor aggressiveness and chemoresistance [8]. In hypoxic conditions, not all the tumor cells are proliferating within the tumor tissue. Since most existing cancer therapies target proliferating cells, a population of non-proliferating cells in tumors could be a source of resistance to therapies or recurrence after therapies. Cells adapt to hypoxic condition by hypoxia-inducible factors (HIFs), which are transcriptional activators and function as master regulators of oxygen homeostasis [9]. However, adaptation to hypoxic conditions requires many complex genetic and biochemical reactions that regulate one another [8].

Micro-ribonucleic acids (miRNAs) are recently discovered small non-coding endogenous RNAs of about 22 nucleotides in length. miRNAs function in RNA silencing and post-transcriptional regulation of gene expression [10] and contribute, as master regulators, of several biological processes, including cell proliferation, differentiation, and metabolism in normal cells [10]. In addition, aberrant miRNAs expression is found in various human cancers [11, 12]. These miRNAs are called “oncomirs” and are associated with tumorigenesis, malignant transformation, metastasis, and chemoresistance. Oncomirs can act as either oncogenes or tumor suppressors. Recent genome-wide approaches suggest that miRNAs differentially expressed in normal and cancer tissues can be direct therapeutic tools or predictive markers for diagnosis, prognosis or therapeutic outcomes in cancer treatment [13].

Angiogenesis is demonstrated to be critical for both tumor formation and progression [14]. Despite active angiogenesis during tumorigenesis, tumor vessels are very irregular, leaky and function poorly [14]. These features can lead to stabilization of hypoxic domains and HIF-α [14]. Hypoxia and HIF pathway activation in tumor cells is a significant stimulus for blood vessel formation and can affect on tumor biology. Indeed, miR-210 becomes progressively upregulated in response to HIFs in hypoxic conditions [15]. In addition, we previously reported that miR-382 induced by hypoxia and HIF-1 promotes angiogenesis and acts as an angiogenic oncogene by repressing phosphatase and tensin homolog (PTEN) [16]. Recently, a subset of cellular miRNAs has been shown to be upregulated during hypoxia, suggesting its involvement in promoting tumor development. This emphasizes the importance of hypoxia-induced miRNAs as targets of tumor progression. miR-210 has been identified as a hypoxia-induced miRNA that plays key roles in biological processes such as cell cycle progression, metabolism, apoptosis, angiogenesis and in the metastasis of cancer [17]. The hypoxic tumor microenvironment correlated with poor response to radiotherapy and chemotherapy [18]. We recently found that miR-382 is upregulated by HIF-1α under hypoxic conditions and increases angiogenesis during tumor growth. In addition, miR-382 increases endothelial cell proliferation, migration, tube formation, and chick embryo angiogenesis. Through PTEN, miR-382 activates the phosphatidylinositol 3-kinase (PI3K)/AKT signaling pathway. Nevertheless, there have been very few studies of hypoxia-induced miRNAs in GCs, and the clinical implications of miRNAs in patients with GC have not been fully elucidated.

This study was designed to analyze the expression of hypoxia-induced miR-210 and miR-382 in gastric adenocarcinoma (STAD) samples as well as big data portal, and to evaluate their relationships with tumor clinicopathologic variables for diagnosis and prognosis.

## Materials and methods

### Ethics approval and consent to participate

The biospecimens for this study were provided by National Biobank of Korea-Kyungpook National University Hospital (KNUH), which is supported by the Ministry of Health, Welfare, and Affairs. All materials derived from the National Biobank of Korea-KNUH were obtained (with informed consent) under institutional review board (IRB)-approved protocols (Title: Expression profile and tumorigenic potential of micoRNAs in stomach cancer tissues, No. KNUMCBIO_14–1014).

### Patients, data, tissue specimen collection, and tissue microarray construction

In this retrospective study, we included 183 formalin-fixed, paraffin-embedded (FFPE) primary gastric adenocarcinoma samples. Surgically resected samples were randomly selected from the 398 archives of the Department of Pathology, Kyungpook National University between January and December 2008. Among these 183 patients, expression of miRNAs was examined in 183 tumor tissues and 60 paired normal mucosal tissues from available FFPE tissue samples. Tissue microarrays from the 183 patients were constructed from the representative areas of individuals paraffin blocks as previously described [19]. All patients were neoadjuvant chemo- or radio therapy naïve. Most patients included in this study were treated by curative intent D2 gastrectomy followed by 5-FU based chemotherapy according to TNM stage and had been followed-up regularly. All data were obtained by reviewing medical records, pathology reports, and hematoxylin and eosin (H&E)-stained sections by two specialized gastrointestinal pathologists (A.N.S and H.I.B). The specific data collected were: age at the time of diagnosis, sex, tumor size, histology grade, lymphovascular invasion, venous invasion, perineural invasion, and pathologic stage. OS of patients was calculated from the time of surgery to death from any cause, as applicable. This study was approved by the Institutional Review Board of Kyungpook National University Chilgok Hospital (2014-04-214). The participants did not provide written informed consent in this study. The Institutional Review Board waived the need for written informed consent under the condition of anonymization and no additional intervention to the participants.

### RNA isolation, miRNA reverse transcription, and real-time qPCR

Three to five serial sections (5 μm thick) from each representative FFPE block were cut, and tumor areas with 80% or more tumor cells were marked and manually dissected. Total RNA was extracted from dissected tissues using the miRNeasy Mini Kit (Qiagen, Valncia, CA, USA) according to the manufacturer’s instructions. The quantity and quality of the isolated total RNA were measured using a NanoDrop^TM^ 2000/2000^c^ spectrophotometer (Thermo Fisher Scientific, Seoul, Republic of Korea). The expressions of miR-210 and miR-382 were analyzed using real time-qPCR (Bio-rad C1000 Touch^TM^ Thermal Cycler, Hercules, USA) using SYBR Green PCR Master Mix (Qiagen, Hilden, Germany) and specific primers for miScript miR-210 (#MS00003801, Qiagen) and miR-382 (#MS00004123, Qiagen) primer assay kit. RNU6P (#MS00033740, Qiagen), RNU44 (#MS00007518, Qiagen), and RNU48 (#MS00007511, Qiagen) primer assay kits were used as endogenous controls for data normalization. Briefly, 10 ng of total RNA was reverse transcribed and complementary DNA used for a quantitative real-time polymerase chain reaction (qPCR), where each experiment was performed in triplicate, and an average was calculated. The difference between the groups was determined by comparative ΔCt, which indicates the difference between the Ct value for the miR of interest and the Ct value for the miRNA used for normalization.

### Patient cohort and miRNAs data from data portal

The miRNA expression data of the gastric adenocarcinoma patients were obtained from the Genomic Data Commons (GDC) data portal, NCI (USA) (https://portal.gdc.cancer.gov/). We downloaded these data on April 3, 2018. These GDC data included 446 gastric adenocarcinoma and 45 normal tissues. We used data from 434 gastric adenocarcinoma tumor samples and 41 adjacent normal tissue samples.

### Statistical analyses

All statistical analyses were performed using SSPS version 18.0 for Windows (SPSS Inc., Chicago, IL, USA). The comparisons of the miRNA expression, shown as the 2^-ΔΔCT^ values, between normal tissues and gastric adenocarcinoma tissues were determined using the Wilcoxon signed rank test. For statistical reasons, receiver-operating characteristic (ROC) curve analysis against OS was performed to determine the clinically relevant miR-210 and miR-382 expression cutoff points. Expression levels of each miRNA were classified as a dichotomous covariate (low or high) according to cutoff point. The categorical variables were analyzed using χ^2^, Fisher’s exact, or McNemar tests, as appropriate. Pearson correlation (*R*) test was used to assess the correlation between miR-210 and miR-382 expression, and correlation between PD-L1 level, CD3+ or CD4+ T cells and miR-210/miR-382 expression level. The statistical comparisons of differentially expressed miRNAs between cancer and non-cancer tissues of stomach adenocarcinoma from the GDC data portal were performed using the Student t test in R package version 3.5.1. Survival curves were plotted using the Kaplan-Meier method, and the significance of differences between groups was calculated using the log-rank test. Multivariate survival analyses were performed using the Cox proportional hazards regression model, using the enter model, after controlling for variables that were proven to significantly be associated with prognosis in univariate analyses. The hazard ratio (HR) and 95% confidence intervals (CI) were assessed for each factor. All statistical tests were two-tailed, with a significance level of 5%.

## Results

### Patient characteristics

A total of 183 patients with surgically resected gastric adenocarcinoma were analyzed and patient demographic and clinic-pathologic characteristics summarized (**S1 Table**). The median age at the time of diagnosis was 63 years (range, 26 to 84 years), 135 (73.8%) were male. The pathologic TNM stage of each tumor was newly determined based on the 7^th^ edition of the Union for International Cancer Control/American Joint Committee on Cancer (UICC/AJCC) for the stomach [20]. At the time of initial resection, 99 (54.1%) tumors were stage I, 55 (30.1%) were stage II, and 29 (15.8%) were stage III.

### miR-210 and miR-382 expression in tumor tissues and the association with clinicopathologic features

We investigated the expression of miR-210 and miR-382, and their association with clinicopathologic parameters in 183 gastric adenocarcinoma tumor tissues. The median level of miR-210 and miR-382 expression was 0.95 (0.15–23.04) and 0.97 (range, 0.17–58.28), respectively. Using ROC curve analysis to predict OS, cutoff values were selected, and confirmed using the maximum χ^2^ test (minimum *p*-value). The most predictive cutoff values for miR-210 and miR-382 were 1.37 and 2.16, respectively. miR-382 expression was significantly correlated with miR-210 expression (*R* = 0.516, *p*<0.001). According to these cutoff values, patients were classified into high expression or low expression groups. Among a total of 183 patients, 53 (29.0%) had high miR-210 and 20 (10.9%) had high miR-382 expression. High miR-210 and miR-382 expression was significantly correlated with unfavorable prognosis including advanced GCs (AGC), higher T category, N category, pathologic TNM stage, lymphovascular invasion, venous invasion, and perineural invasion, respectively (**Table 1**). Lymphovascular, venous and perineural invasion has been associated with poor prognosis in many solid tumors [21–24] because the lymphovascular spaces, venous circulation and nerve microenvironment may be the routes of metastatic spread. Therefore, these results suggest that hypoxamiRs, miR-210 and miR-382 can be associated with adverse pathological features for GCs, such as elevated recurrence rate and diminished survival.

**Table 1:** Relationship between clinicopathologic characteristics of tumor and expression levels of miR-382 and miR-210 in gastric adenocarcinoma.

Clinicopathological		microRNA-382		*p*	microRNA-210		*p*
Characteristics	All	Low expression	High expression		Low expression	High expression	
	*n* (%)	*n* (%)	*n* (%)		*n* (%)	*n* (%)	
Age at diagnosis, years				0.349			0.034
<63	90 (49.2)	78 (47.9)	12 (60.0)		71 (54.2)	19 (36.5)	
≥63	93 (50.8)	85 (52.1)	8 (40.0)		60 (45.8)	33 (63.5)	
Sex				0.788			0.854
Male	135 (73.8)	121 (74.2)	14 (70.0)		95 (73.1)	40 (75.5)	
Female	48 (26.2)	42 (25.8)	6 (30.0)		36 (26.9)	13 (24.5)	
Lauren classification				0.663			0.937
Intestinal	67 (36.6)	61 (37.4)	6 (30.0)		48 (36.9)	19 (35.8)	
Diffuse	101 (55.2)	88 (54.0)	13 (65.0)		72 (55.4)	29 (54.7)	
Mixed	15 (8.2)	14 (8.6)	1 (5.0)		10 (7.7)	5 (9.4)	
EGC^a^ vs AGC^b^				**0.008**			**<0.001**
EGC	91 (49.7)	87 (53.4)	4 (20.0)		81 (61.8)	10 (19.2)	
AGC	92 (50.3)	76 (46.6)	16 (80.0)		50 (38.2)	42 (80.8)	
Pathologic T category				**<0.001**			**<0.001**
pT1-T2	114 (62.3)	109 (66.9)	5 (25.0)		99 (75.6)	15 (28.8)	
pT3-T4	69 (37.7)	54 (33.1)	15 (75.0)		32 (24.4)	37 (71.2)	
Pathologic N category				**0.023**			**<0.001**
pN0	125 (68.3)	116 (71.2)	9 (45.0)		102 (77.9)	23 (44.2)	
pN1-3	58 (31.7)	47 (28.8)	11 (55.0)		29 (22.1)	29 (55.8)	
Pathologic stage				**<0.001**			**<0.001**
I	99 (54.1)	95 (58.3)	4 (20.0)		86 (85.6)	13 (25.0)	
II	55 (30.1)	48 (29.4)	7 (35.0)		34 (26.0)	21 (40.4)	
III	29 (15.8)	20 (12.3)	9 (45.0)		11 (8.4)	18 (34.6)	
Lymphovascular invasion				**0.002**			**0.001**
Negative	130 (71.0)	122 (74.8)	8 (40.0)		102 (78.5)	28 (52.8)	
Positive	53 (29.0)	41 (25.2)	12 (60.0)		28 (21.5)	25 (47.2)	
Venous invasion				**0.001**			**0.008**
Negative	177 (96.7)	161 (98.8)	16 (80.0)		129 (99.2)	48 (90.6)	
Positive	6 (3.3)	2 (1.2)	4 (20.0)		1 (0.8)	5 (9.4)	
Perineural invasion				**<0.001**			**0.047**
Negative	160 (87.4)	149 (91.4)	11 (55.0)		118 (90.8)	42 (79.2)	
Positive	23 (12.6)	14 (8.6)	9 (45.0)		12 (9.2)	11 (20.8)	

^a^ Early gastric carcinoma;

^b^ Advanced gastric carcinoma.

### Patient outcome

**Fig 1:**
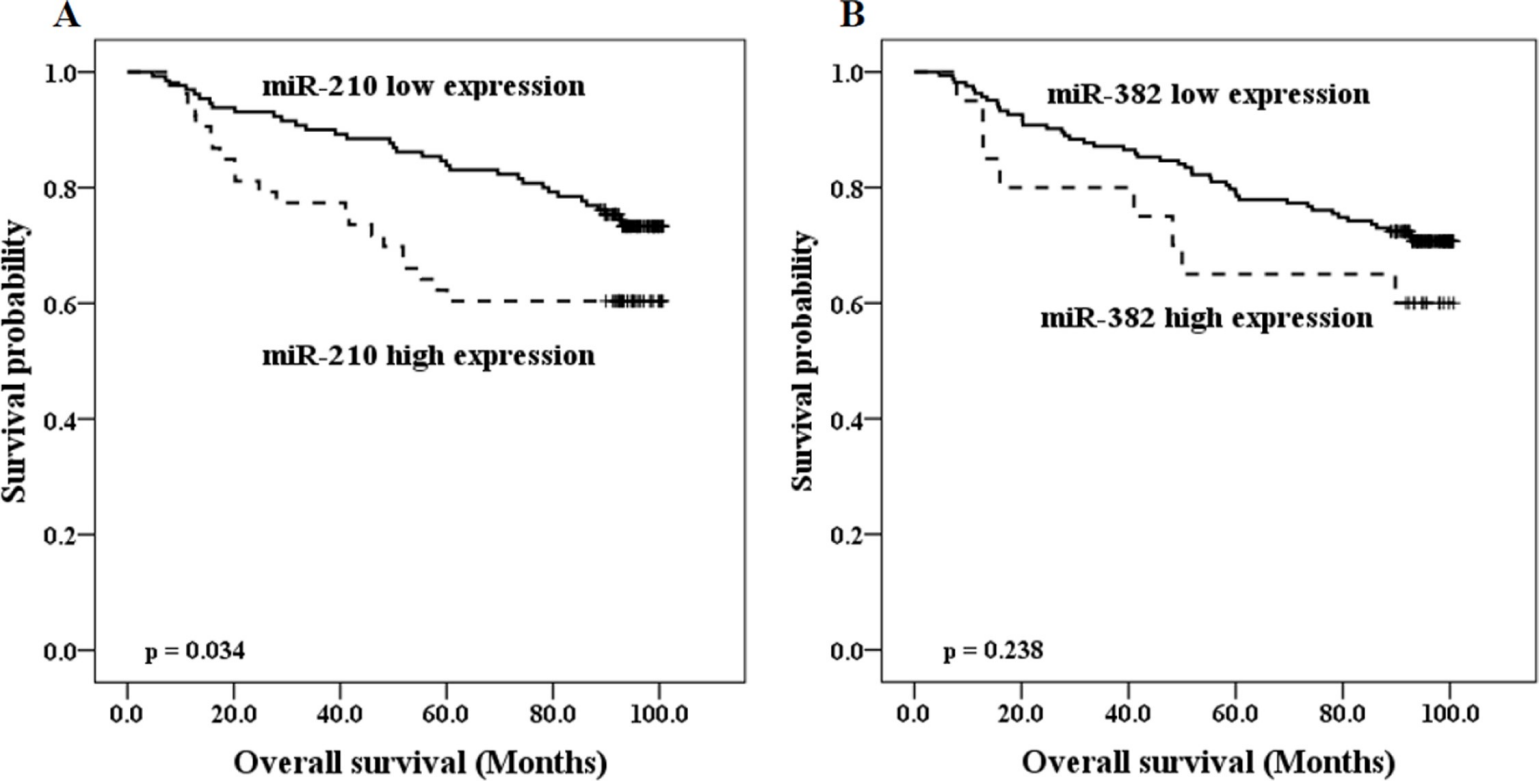
Prognostic values of miR-210 and miR-382 expression in GC patients. Estimated Kaplan-Meyer curves of overall survival for (A) miR-210 and (B) miR-382 in 183 gastric cancer (GC) patients.

At the time of the primary analysis (April 2016), the median follow-up period was 93 months (range, 4.7 to 100.7 months). In this period, 55 (30.1%) patients died. In univariate analysis, patients with high miR-210 had significantly shorter OS than did those with low miR-210 expression (*p* = 0.034, **Fig 1A**). However, the duration of OS of low and high miR-382 expression groups did not differ significantly (*p* = 0.238, **Fig 1B**). In a Cox proportional hazard model adjusted for age, lymphovascular invasion, perineural invasion, T category, and N category, high miR-210 expression was not independently associated with OS in patients with gastric adenocarcinoma (*p* = 0.185, HR = 0.655, 95% CI = 0.351–1.223; **Table 2**). From these results, we raised a question whether these two hypoxa-miRs have different correlations in the progression of GC subgroups.

**Table 2:** Univariate and multivariate survival analysis for overall survival in gastric adenocarcinoma.

Variable	Category	Overall survival			
		Univariate analysis		Multivariate analysis	
		*p*	HR^a^ (95% CI^b^)	*p*	HR (95% CI)
Age	<63 *vs* ≥63	<0.001	2.757 (1.540–4.936)	0.010	2.192 (1.205–3.989)
Sex	Male *vs* Female	0.120	0.571 (0.279–1.168)	—	—
pT category	T1/T2 *vs* T3/T4	<0.001	5.484 (3.087–9.745)	<0.001	4.817 (2.463–9.422)
pN category	N0 *vs* N1-N3	<0.001	3.192 (1.876–5.431)	0.245	1.517 (0.751–3.063)
Lymphatic invasion	Absent *vs* Present	<0.001	2.811 (1.654–4.777)	0.333	1.387 (0.715–2.691)
Venous invasion	Absent *vs* Present	0.790	1.211 (0.295–4.969)	—	—
Perineural invasion	Absent *vs* Present	0.002	2.614 (1.376–4.965)	0.850	0.936 (0.471–1.860)
miR-210 expression	Low *vs* High	0.034	1.853 (1.074–3.194)	0.162	0.644 (0.347–1.194)
miR-382 expression	Low *vs* High	0.238	1.564 (0.739–3.311)	—	—

^a^ Hazard ratio;

^b^ Confidence interval; vs, versus.

### Subset analysis regarding Lauren classification

**Fig 2:**
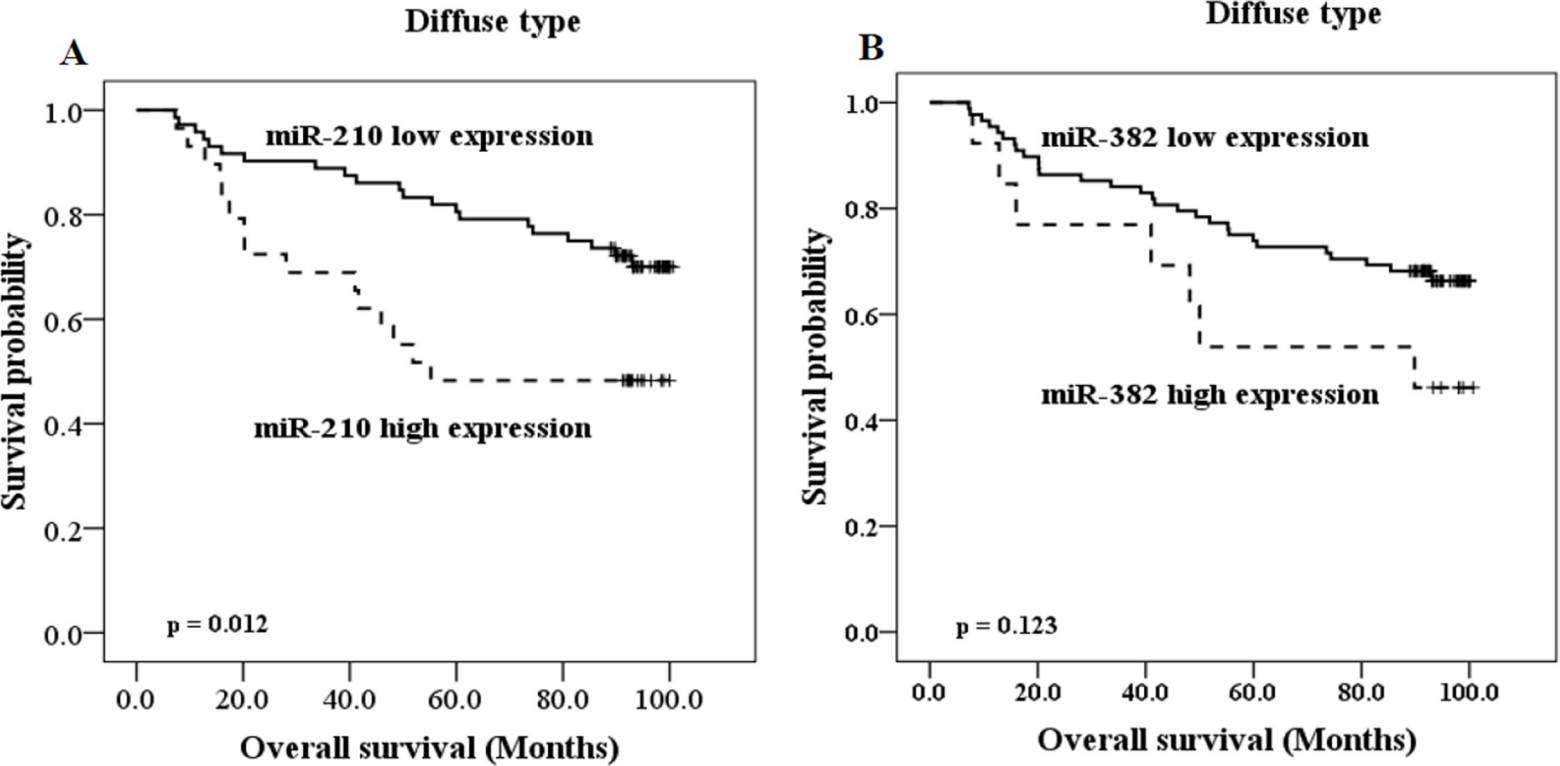
Prognostic values of miR-210 and miR-382 expression in subgroup analysis according to Lauren classification diffuse type GC. Estimated Kaplan-Meyer curves of overall survival for (A) miR-210 and (B) miR-382 in 101 patients with diffuse type GC.

Next, we performed subgroup analyses according to Lauren classification, because intestinal and diffuse GCs display numerous pathological, epidemiological and etiological differences. Investigation of clinical significance of miR-210 and miR-382 in patients group with intestinal type GC (*n* = 67) showed that high miR-210 and miR-382 expression correlated with AGC, higher T category, N category, and pathologic stage, lymphovascular invasion, venous invasion, and perineural invasion, respectively (**Table 3**). On the other hand, in patients group with diffuse type GC (*n* = 101), high miR-210 expression was associated with old age, AGC, higher T category, N category, and pathologic stage (**Table 4**). High miR-382 expression was related to higher T category, pathologic stage, and perineural invasion (**Table 4**). However, as there were as only a small number of patients with mixed type (*n* = 15), we could not determine the clinical significance of miR-210 and miR-382 expression in this subgroup. High miR-210 expression was significantly associated with poor OS in the patient group with diffuse type GC (*p* = 0.012, **Fig 2A**), but not intestinal type GC (*p* = 0.477, data no shown). In contrast, high miR-382 expression showed only a tendency of the association with worse OS in patient group with diffuse type GC, but statistical significance was not reached (*p* = 0.123, **Fig 2B**). The results suggest that expression of miR-210 and miR-382 is more correlated with clinical outcomes of GCs of intestinal type than those of diffuse type, but more correlated with poor prognosis of diffuse type GCs than intestinal type GCs. However, we can suggest from these results obtained by the limited tissues that significant correlation with OS for miR-382 in diffuse type GCs has to be further identified using a greater number of patients.

**Table 3:** Relationship between clinicopathologic characteristics of tumor and expression levels of miR-382 and miR-210 in gastric adenocarcinoma with intestinal type of Lauren classification.

		Intestinal type					
Clinicopathological	All	microRNA-382		*p*	microRNA-210		*p*
Characteristics	*n* (%)	Low expression	High expression		Low expression	High expression	
		*n* (%)	*n* (%)		*n* (%)	*n* (%)	
Age at diagnosis, years				0.197			1.000
<63	26 (38.8)	22 (36.1)	4 (66.7)		19 (39.6)	7 (36.8)	
≥63	41 (61.2)	39 (63.9)	2 (33.3)		29 (60.4)	12 (63.2)	
Sex				0.639			0.538
Male	50 (74.6)	46 (75.4)	4 (66.7)		37 (77.1)	13 (68.4)	
Female	17 (25.4)	15 (24.6)	2 (33.3)		11 (22.9)	6 (31.6)	
EGC^a^ vs AGC^b^				**0.013**			**0.002**
EGC	45 (67.2)	44 (72.1)	1 (16.7)		38 (79.2)	7 (36.8)	
AGC	22 (32.8)	17 (27.9)	5 (83.3)		10 (20.8)	12 (63.2)	
Pathologic T category				**0.032**			**<0.001**
pT1-T2	50 (74.6)	48 (78.7)	2 (33.3)		42 (87.5)	8 (42.1)	
pT3-T4	17 (25.4)	13 (21.3)	4 (66.7)		6 (12.5)	11 (57.9)	
Pathologic N category				**0.011**			**0.038**
pN0	54 (80.6)	52 (85.2)	2 (33.3)		42 (87.5)	12 (63.2)	
pN1-3	13 (19.4)	9 (14.8)	4 (66.7)		6 (12.5)	7 (36.8)	
Pathologic stage				**0.004**			**<0.001**
I	47 (70.1)	46 (75.4)	1 (16.7)		40 (83.3)	7 (36.8)	
II	16 (23.9)	13 (21.3)	3 (50.0)		7 (14.6)	9 (47.4)	
III	4 (6.0)	2 (3.3)	2 (33.3)		1 (2.1)	3 (15.8)	
Lymphovascular invasion				**0.005**			**0.009**
Negative	56 (83.6)	54 (88.5)	2 (33.3)		44 (91.7)	12 (63.2)	
Positive	11 (16.4)	7 (11.5)	4 (66.7)		4 (8.3)	7 (36.8)	
Venous invasion				**0.020**			**0.020**
Negative	64 (95.5)	60 (98.4)	4 (66.7)		48 (100.0)	16 (84.2)	
Positive	3 (4.5)	1 (1.6)	2 (33.3)		0 (0)	3 (15.8)	
Perineural invasion				**0.004**			**0.020**
Negative	62 (92.5)	59 (96.7)	3 (50.0)		47 (97.9)	15 (78.9)	
Positive	5 (7.5)	2 (3.3)	3 (50.0)		1 (2.1)	4 (21.1)	

^a^ Early gastric carcinoma;

^b^ Advanced gastric carcinoma.

**Table 4:** Relationship between clinicopathologic characteristics of tumor and expression levels of miR-382 and miR-210 in gastric adenocarcinoma with diffuse type of Lauren classification.

		Diffuse type					
Clinicopathological	All	microRNA-382		*p*	microRNA-210		*p*
Characteristics	*n* (%)	Low expression	High expression		Low expression	High expression	
		*n* (%)	*n* (%)		*n* (%)	*n* (%)	
Age at diagnosis, years				0.769			**0.003**
<63	59 (58.4)	52 (59.1)	7 (53.8)		49 (68.1)	10 (34.5)	
≥63	42 (41.6)	36 (40.9)	6 (46.2)		23 (31.9)	19 (65.5)	
Sex				0.639			0.481
Male	71 (70.3)	62 (70.5)	9 (69.2)		49 (68.1)	22 (75.9)	
Female	30 (29.7)	26 (29.5)	4 (30.8)		23 (31.9)	7 (24.1)	
EGC^a^ vs AGC^b^				0.228			**<0.001**
EGC	42 (41.6)	39 (44.3)	3 (23.1)		38 (52.8)	4 (13.8)	
AGC	59 (58.4)	49 (55.7)	10 (76.9)		34 (47.2)	25 (86.2)	
Pathologic T category				**0.016**			**0.001**
pT1-T2	56 (55.4)	53 (60.2)	3 (23.1)		48 (66.7)	8 (27.6)	
pT3-T4	45 (44.6)	35 (39.8)	10 (76.9)		24 (33.3)	21 (72.4)	
Pathologic N category				0.360			**0.003**
pN0	62 (61.4)	56 (63.6)	6 (46.2)		51 (70.8)	11 (37.9)	
pN1-3	39 (38.6)	32 (36.4)	7 (53.8)		21 (29.2)	18 (62.1)	
Pathologic stage				**0.013**			**0.006**
I	46 (45.5)	43 (48.9)	3 (23.1)		39 (54.2)	7 (24.1)	
II	34 (33.7)	31 (35.2)	3 (23.1)		23 (31.9)	11 (37.9)	
III	21 (20.8)	14 (15.9)	7 (53.8)		10 (13.9)	11 (37.9)	
Lymphovascular invasion				0.113			0.108
Negative	68 (67.3)	62 (70.5)	6 (46.2)		52 (72.2)	16 (55.2)	
Positive	33 (32.7)	26 (29.5)	7 (53.8)		20 (27.8)	13 (44.8)	
Venous invasion				0.242			1.000
Negative	99 (98.0)	87 (98.9)	12 (92.3)		71 (98.6)	28 (96.6)	
Positive	2 (2.0)	1 (1.1)	1 (7.7)		1 (1.4)	1 (3.4)	
Perineural invasion				**0.041**			0.561
Negative	84 (83.2)	76 (86.4)	8 (61.5)		61 (84.7)	23 (79.3)	
Positive	17 (16.8)	12 (13.6)	5 (38.5)		11 (15.3)	6 (20.7)	

^a^ Early gastric carcinoma;

^b^ Advanced gastric carcinoma.

### Differential expression pattern of miR-210 and miR-382 in normal and tumor tissues with paired samples as well as big data analysis in gastric adenocarcinoma patients

**Fig 3:**
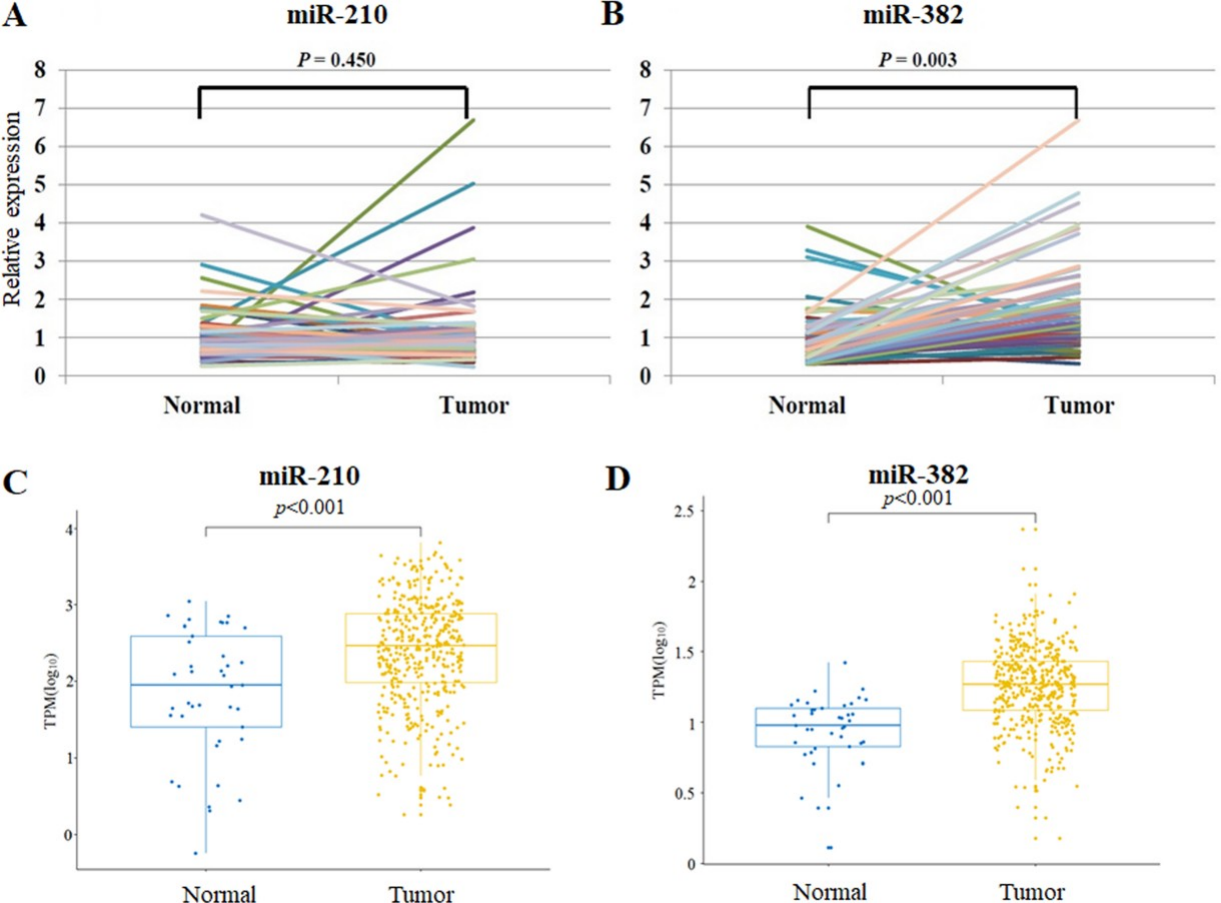
Predictive values of miR-210 and miR-382 expression in gastric adenocarcinoma in comparison with normal tissues. (A-B) The levels of miR-210 and miR-382 were measured by quantitative PCR after reverse transcription. miR-382 expression was significantly higher in tumor than that in paired normal tissues (*p*<0.001), while miR-210 expression did not differ between two groups (*p* = 0.472). (C-D) GDC data set analysis of miR-210 and miR-382 expression was performed. 446 GCs (STAD) and 45 normal tissue was analyzed using the GDC data set. P value is calculated by using Student t test.

To investigate the differential expression pattern of miR-210 and miR-382 in tumor tissues with normal tissues, we evaluated the miR-210 and miR-382 expression in 60 randomly selected patients among 183 cases with gastric adenocarcinoma and in 60 paired normal gastric mucosal tissues. The demographics of the 60 patients were provided in **S2 Table**. miR-382 expression was significantly higher in tumor tissues than that in paired controls (*p* = 0.003), while miR-210 expression did not differ between two groups (*p* = 0.450) (**Fig 3**). The results are likely different from the overall survival data in Fig 1. Therefore to have more convincing data, we extend this analysis into the non-paired normal and tumor tissues by increasing the patient number using a big data analysis with 446 gastric adenocarcinoma and 45 normal tissues from the data portal (https://portal.gdc.cancer.gov/). As a result, miR-210 expression of gastric adenocarcinoma significantly increased compared with normal tissues (*p* <0.001). And miR-382 expression of gastric adenocarcinoma significantly increased compared with normal tissues (*p*<0.001). The paired samples might be a more exact comparison in an individual base, but paired samples were limited for the PCR analysis in our resources and limited number cannot ensure the general investigation. Thus, after we increased patient samples even not with paired samples using a big data, we obtained the results that miR-210 as well as miR-382 expression was significantly higher than the normal tissues.

Recent reports have suggested that upregulation of programmed death-ligand 1 (PD-L1) in cancer is widely implicated in tumor immune evasion and is highly associated with tumor aggressiveness, which limits the effectiveness of anti-cancer agents [25]. Thus, we tried to find correlation of miR-210/miR-382 levels with immune cell infiltration degree as well as PD-L1 status in the gastric cancer patients. Using TMA of 183 gastric cancer patients and IHC analysis, we identified infiltrating CD3+ (TIL), CD4+ regulatory and CD8+ cytotoxic T cells. However, we could not find significant correlation between CD3+, CD4+ T cell number and expression level of our hypoxamiRs except for miR210 and CD4+ cells (S1 Fig). Unfortunately, we scarcely detected CD8+ T cells in the gastric cancer tissues, thus we could not analyze the correlation. In addition, correlation between PD-L1 level and expression level of our hypoxamiRs was not significant (S2 Fig).

Taken together, we can conclude that hypoxa-miRs, miR-210 and miR-382 may play important roles in aggressiveness, progression and prognosis of GCs, but miR-382 would be provided more as a predictive marker for progression of GCs from the normal or preneoplastic lesion.

## Discussion

Hypoxia is a common causative feature for pathological conditions such as tissue ischemia, inflammation and solid tumorigenesis. Recently, it has been revealed that a specific set of miRNA molecules are upregulated under hypoxic conditions. We conducted this study to determine whether expression levels of these “hypoxamiRs” miR-210 and miR-382 is associated with clinicopathologic parameters and their impact on survival in patients with operable GC. To the best of our knowledge, this is the first report studying expression of miR-210 and miR-382 in East Asians with GC. High miR-210 expression has a negative, but not independent prognostic impact in patients with surgically resected GC. High miR-210 and miR-382 expression correlated with categories related to unfavorable prognosis including higher stage, lymphovascular invasion, venous invasion, and perineural invasion.

In 2007, Kulshreshtha et al. first identified hypoxic regulation of miR-210 by miRNA microarray [26]. miR-210 is a unique miRNA that is consistently up-regulated by both HIF-1α and HIF-2α, and plays an important role in the cellular response to hypoxia [27]. Several studies since then have confirmed that miR-210 is frequently over-expressed in various cancers likely owing to the occurrence of hypoxia inside solid tumors [27, 28]. High miR-210 expression is associated with poor prognosis in patients with breast and pancreatic cancers. In particular, miR-210 was found to be highly expressed in triple-negative breast cancer (TNBC) both in tumor cells and in the tumor microenvironment [29]. Interestingly, Kiga *et al*. [28] suggested that a decrease in miR-210 expression could be a risk factor for development of H. pylori-associated gastric diseases and GC. Using a microRNA microarray assay, they showed that miR-210 expression is significantly higher in normal regions, than paired tumor regions of FFPE specimens biopsied from 20 GC patients [28]. In contrast, Zhou el al. showed that miR-210 was significantly up-regulated in the peripheral plasma of GC patients compared to normal controls [30]. In our study, miR-382 expression was found to be significantly higher in tumor tissues than in paired normal tissues, but miR-210 expression did not differ between two groups of 60 FFPE specimens from GC patients. Thus, increased number of GC patients or big data analysis should be adopted for delineating the correlation of GC progression with expression of miR210 and miR-382. Limited amount of the normal compartment in patient tissues was our big obstacle in paired analysis.

miR-382 exists in a miRNA cluster in the imprinted DLK1-DIO3 region on the 14q32 locus, which hosts one of the largest miRNA clusters in the human genome [31]. The studies have shown that miR-382 was down-regulated or up-regulated in several cancers. Some studies have reported that downregulation of miR-382 was associated with poor patient outcome for patients with esophageal squamous cell carcinoma [31], and osteosarcoma [32]. Thereby, they suggested that miR-382 is a tumor suppressor miRNA and induction of miR-382 is a potential strategy to inhibit tumor cell progression and chemoresistance [31, 32]. In contrast, Ho et al. demonstrated that up-regulation of miR-382 was a risk factor for breast cancer carcinogenesis as an oncomiR and is an independent, poor prognostic factor for OS and DFS in patients with breast cancer [33]. Similarly, our previous study showed that miR-382 was a strong oncomiR and hypoxamiR in gastric cancer cells which induces angiogenic phenotypes in ECs, suggesting that metastatic potential can be induced by miR-382 in GCs [16]. In the present study, miR-382 was up-regulated in GC and is associated with higher stage, lymphovascular invasion, venous invasion, and perineural invasion. These findings could support the role of miR-382 as an oncomiR in GC. Furthermore, in our previous study, we suggested that miR-382 may critically increase angiogenesis by inducing VEGF via HIF-1α, which is mediated by the AKT/mTOR signaling pathway, and could be inhibited by PTEN [16]. Although further studies on the biological role of miR-382 as a hypoxamiR and its relationship with HIF-1α are required, the present study showed a significantly positive correlation between miR-382 and miR-210 expressions as representative hypoxamiRs.

Rotkrua et al. revealed that miR-103, miR-107, miR-194, and miR-210 were significantly higher in the serum of early-stage diffuse type GC than in healthy controls. They suggested that these miRNAs in serum could be novel non-invasive biomarkers for the early detection of diffuse type GC [34]. They also demonstrated that miR-103 and miR-194 levels in the serum up-regulated in a stepwise manner during the progression from normal to early-stage diffuse type and finally to advanced-stage diffuse type GC, while, miR-210 expression level in the serum was significantly reduced during development from early-stage diffuse type to advanced-stage diffuse type GC [34]. However, in the present study, high miR-210 expression was significantly associated with both intestinal type and diffuse type AGCs in FFPE specimens. This inconsistency may be caused by a difference between circulating miR-210 in serum and miR-210 expression within tumors of FFPE specimen. Because miRNAs are short-stranded RNAs and are well preserved for long periods and at high temperatures, miRNAs can be easily detected in FFPE specimen [35]. miR-210, in particular, is known to be the most robustly induced miRNA under hypoxic conditions [36], and may be a surrogate marker for tumor hypoxia [27]. Further studies examining the realistic possibility of miR-210 as a novel biomarker for non-invasive detection of GC are necessary.

So far, the development of miRNA-targeting therapies for clinical practice has been highlighted. The current clinical studies are focused on anti-miRNA oligonucleotides or anti-miRNAs, including antagomirs, miRNA-sponges, miRNA-masks, locked nucleic acid (LNAs) probes, peptide nucleic acid (PNA) probes, and siRNA, all of which could target oncogene-specific miRNAs [17]. Future studies are warranted to explore potential methods for targeting miR-210 and miR-382 as therapeutic tools.

Our study was limited because of its retrospective nature, use of a single institution, and random selection bias. In addition, the relatively small sample size precludes robust statistical analysis. However, we supplemented the sample size with big data from portal and obtained better clinically significant meaning for the two miRs. Finally, characteristics of miRNAs that partially or perfectly match target mRNA 3’UTR region sequences are critical for the mechanism of their action. Having multiple targets for one miRNA limited our research. Despite these limitations, miRNAs are useful tools for diagnosis, prognosis, and therapeutics because detecting the expression of miRNAs is relatively easy and simple. The interpretation of our results must keep this in mind and further large-scale studies will be required to validate our findings in an independent cohort, especially in a Western population.

In summary, we demonstrated that high miR-210 and miR-382 expression were related to tumor aggression. Although miR-210 expression is not an independent prognostic factor, it can be used to predict a poor prognostic outcome. In conclusion, these results show that miR-210 and miR-382 play a significant role in the progression and prognosis of GCs. Furthermore, miR-210 and miR-382 could be relevant therapeutic molecular targets for patients with GCs.

## Supporting information

S1 Method Immunohistochemistry (IHC). (DOCX)

S1 Fig Correlation between CD3+/CD4+ T cell number and expression of miR-210 or miR-382. (DOCX)

S2 Fig Correlation between PD-L1 status and miR-210 or miR-382 expression. (DOCX)

S1 Table Baseline characteristics of 183 patients. (DOCX)

S2 Table Baseline characteristics of 60 patients. (DOCX)
