# Supplementary material for: Clinical significance and prognostic role of hypoxia-induced microRNA 382 in gastric adenocarcinoma
Source: PLoS One. 2019 Oct 9;14(10):e0223608. doi: 10.1371/journal.pone.0223608 (PMC6785122; doi:10.1371/journal.pone.0223608)
Supplement: S1 Method — (DOCX) [file pone.0223608.s001.docx]

**S1 Method**

**Immunohistochemistry (IHC)**

Paraffin-embedded tissue microarray sections (4 µm) were deparaffinized and hydrated for IHC. Briefly, after the slides were blocked with 3% bovine serum albumin in PBS at RT for 1 h and incubated with anti-CD3 (Abcam), PE conjugated anti-CD4 (RM4-5) (Invitrogen), and Alexa Fluor^®^ 647-conjugated anti-CD8a (BioLegend), and anti-PD-L1 antibodies (Cell Signaling) at 4°C overnight. Corresponding secondary antibodies were treated for 2 h and mounted in Antifade Mounting Medium with DAPI (Vectashield). Fluorescence images were acquired using Axio Imager A1 microscope (Carl Zeiss, Germany) and quantified using ImageJ program.
