# Supplementary material for: Clinical significance and prognostic role of hypoxia-induced microRNA 382 in gastric adenocarcinoma
Source: PLoS One. 2019 Oct 9;14(10):e0223608. doi: 10.1371/journal.pone.0223608 (PMC6785122; doi:10.1371/journal.pone.0223608)
Supplement: S1 Fig — (DOCX) [file pone.0223608.s002.docx]

**
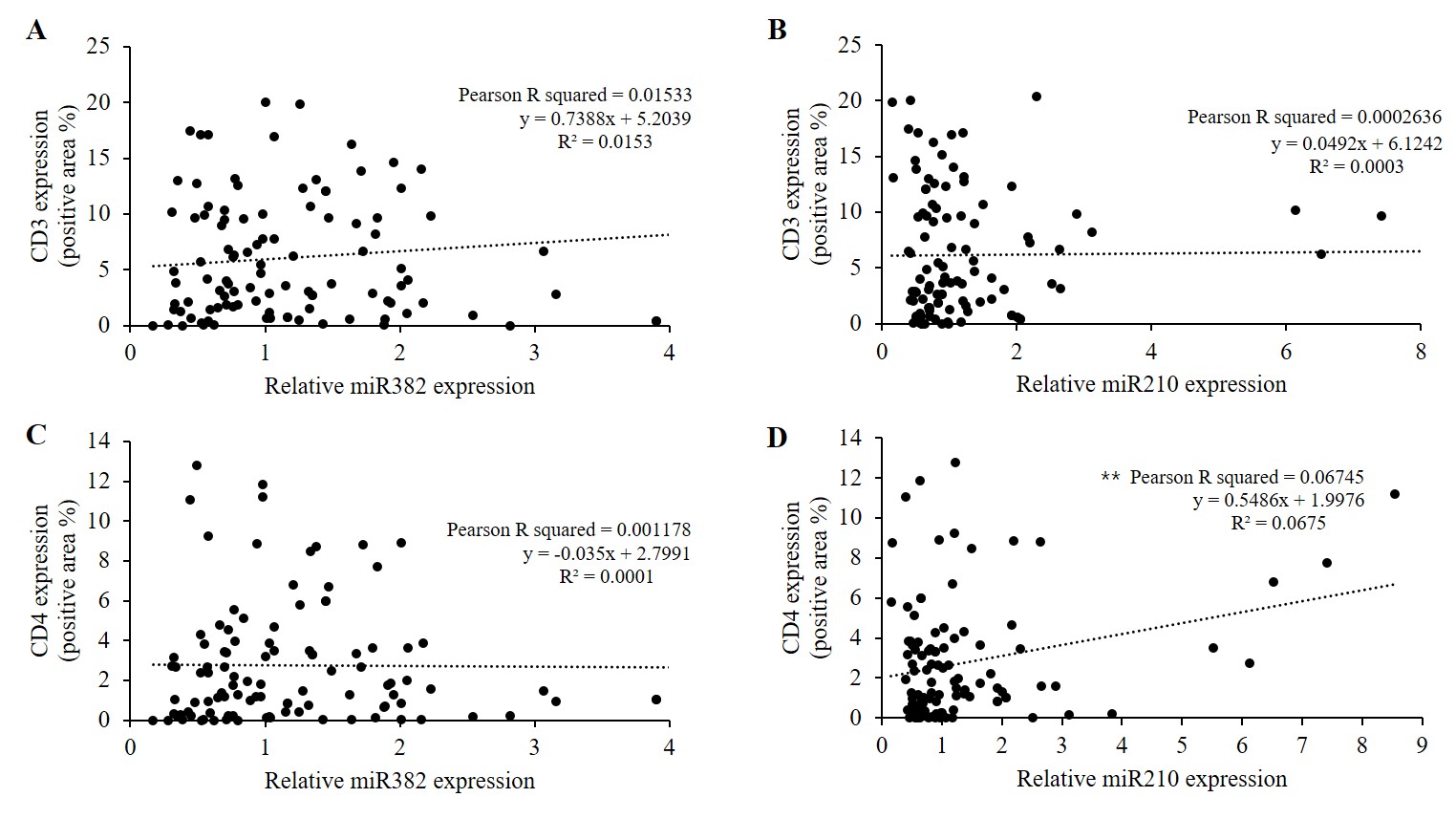
**

**S1 Fig. Correlation between CD3+/CD4+ T cell number and expression of miR-210 or miR-382.** Expression of CD3 (A, B) and CD4 (C,D) was identified by IHC in TMA with 183 gastric cancer patients and analyzed correlation between CD3+/CD4+ area and the hypoxamiRs expression level by Pearson correlation test. Level of microRNAs are shown on the χ-axis, CD3 or CD4 expression levels are shown on the y-axis (significances, A: *P*=0.2061, B: *P*=0.8700, C: *P*=0.9121, D: *P*=0.0075).
