# Supplementary material for: Clinical significance and prognostic role of hypoxia-induced microRNA 382 in gastric adenocarcinoma
Source: PLoS One. 2019 Oct 9;14(10):e0223608. doi: 10.1371/journal.pone.0223608 (PMC6785122; doi:10.1371/journal.pone.0223608)
Supplement: S2 Fig — (DOCX) [file pone.0223608.s003.docx]

**
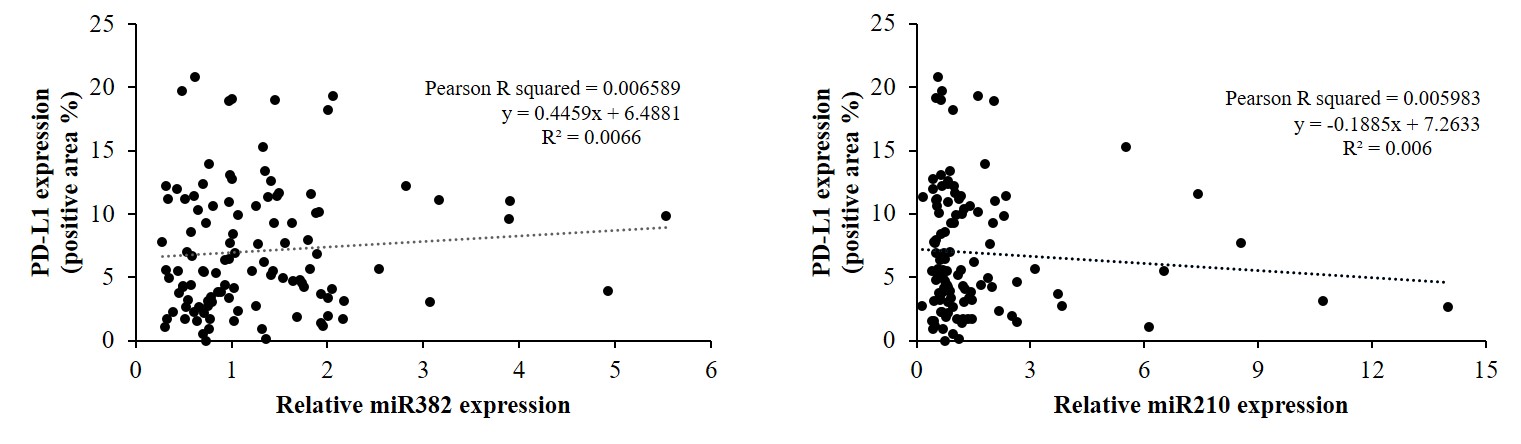
**

**S2 Fig. Correlation between PD-L1 status and miR-210 or miR-382 expression.** Expression of PD-L1 was identified by IHC in TMA with 183 gastric cancer patients and analyzed correlation between PD-L1 status and the hypoxamiRs expression level by Pearson correlation test. Level of microRNAs are shown on the χ-axis, PD-L1 expression levels are shown on the y-axis (significances, left: P=0.3970 and right: P=0.4155).
