# Supplementary material for: Clinical significance and prognostic role of hypoxia-induced microRNA 382 in gastric adenocarcinoma
Source: PLoS One. 2019 Oct 9;14(10):e0223608. doi: 10.1371/journal.pone.0223608 (PMC6785122; doi:10.1371/journal.pone.0223608)
Supplement: S1 Table — (DOCX) [file pone.0223608.s004.docx]

**S1 Table. Baseline characteristics of 183 patients**

| Patient and Disease Characteristic | *N* | % |
| --- | --- | --- |
| Age at diagnosis, years |  |  |
| Median | 63.0 | |
| Range | 26.0-84.0 | |
| Sex |  |  |
| Male | 135 | 73.8 |
| Female | 48 | 26.2 |
| Lauren classification |  |  |
| Intestinal | 67 | 36.6 |
| Diffuse | 101 | 55.2 |
| Mixed | 15 | 8.2 |
| Pathologic T category |  |  |
| T1 | 91.0 | 49.7 |
| T2 | 23 | 12.6 |
| T3 | 43 | 23.5 |
| T4 | 26 | 14.2 |
| Pathologic N category |  |  |
| N0 | 123 | 67.2 |
| N1 | 28 | 15.3 |
| N2 | 16 | 8.7 |
| N3 | 16 | 8.7 |
| Pathologic stage |  |  |
| I | 99 | 54.1 |
| II | 55 | 30.1 |
| III | 29 | 15.8 |
| Lymphovascular invasion |  |  |
| Negative | 130 | 71.0 |
| Positive | 53 | 29.0 |
| Venous invasion |  |  |
| Negative | 177 | 96.7 |
| Positive | 6 | 3.3 |
| Perineural invasion |  |  |
| Negative | 160 | 87.4 |
| Positive | 23 | 12.6 |
