# Supplementary material for: Clinical significance and prognostic role of hypoxia-induced microRNA 382 in gastric adenocarcinoma
Source: PLoS One. 2019 Oct 9;14(10):e0223608. doi: 10.1371/journal.pone.0223608 (PMC6785122; doi:10.1371/journal.pone.0223608)
Supplement: S2 Table — (DOCX) [file pone.0223608.s005.docx]

**S2 Table. Baseline characteristics of 60 patients**

| Patient and Disease Characteristic | *N* | % |
| --- | --- | --- |
| Age at diagnosis, years |  |  |
| Median | 62.5 | |
| Range | 26.0-84.0 | |
| Sex |  |  |
| Male | 41 | 68.3 |
| Female | 19 | 31.7 |
| Lauren classification |  |  |
| Intestinal | 24 | 40.0 |
| Diffuse | 34 | 56.7 |
| Mixed | 2 | 3.3 |
| Pathologic T category |  |  |
| T1 | 36 | 60.0 |
| T2 | 10 | 16.7 |
| T3 | 6 | 10.0 |
| T4 | 8 | 13.3 |
| Pathologic N category |  |  |
| N0 | 43 | 71.7 |
| N1 | 7 | 11.7 |
| N2 | 6 | 10.0 |
| N3 | 4 | 6.7 |
| Pathologic stage |  |  |
| I | 41 | 68.3 |
| II | 10 | 16.7 |
| III | 9 | 15.0 |
| Lymphovascular invasion |  |  |
| Negative | 49 | 81.7 |
| Positive | 11 | 18.3 |
| Venous invasion |  |  |
| Negative | 59 | 98.3 |
| Positive | 1 | 1.7 |
| Perineural invasion |  |  |
| Negative | 53 | 88.3 |
| Positive | 7 | 11.7 |
